# p-21 Activated Kinase as a Molecular Target for Chemoprevention in Diabetes †

**DOI:** 10.3390/geriatrics3040073

**Published:** 2018-10-19

**Authors:** Kyle Dammann, Vineeta Khare, Clyde Coleman, Henrik Berdel, Christoph Gasche

**Affiliations:** 1 Department of Clinical Medicine, Medical University of the Americas, Devens, MA 01434, USA; 2 Department of Internal Medicine III, Medical University of Vienna, Vienna 1090, Austria; vineeta.khare@meduniwien.ac.at (V.K.); christoph.gasche@meduniwien.ac.at (C.G.); 3 Department of Surgery, University of Kentucky HealthCare, Lexington, KY 40536, USA; ccole2@uky.edu; 4 Department of Acute Care and Trauma Surgery, University of Kentucky HealthCare, Lexington, KY 40536, USA; Henrik.Berdel@uky.edu

**Keywords:** p-21 activated kinase, pioglitazone, metformin, type 2 diabetes mellitus, cancer, chemoprevention, inflammation

## Abstract

Hypothesis: Anti-diabetic drugs modulate p-21 activated kinase (PAK) signaling. **Introduction**: Type 2 diabetes mellitus (T2DM) is a chronic inflammatory disease associated with increased cancer risk. PAK signaling is implicated in cellular homeostasis when regulated, and cancer when unrestrained. Recent reports provided a role for PAK signaling in glucose homeostasis, but the role of PAKs in the pathogenesis of T2DM is unknown. Here, we performed a mini-meta-analysis to explore if anti-diabetic drugs modify PAK signaling pathways, and provide insight regarding modulation of these pathways, to potentially reduce diabetes-associated cancer risk. **Methods**: PAK interacting partners in T2DM were identified using the online STRING database. Correlation studies were performed via systematic literature review to understand the effect of anti-diabetic drugs on PAK signaling. A mini-meta-analysis correlated multiple clinical studies and revealed the overall clinical response rate and percentage of adverse events in piogliazone (n = 53) and metformin (n = 91) treated patients with PAK-associated diseases. **Results**: A total of 30 PAK interacting partners were identified (10: reduced beta-cell mass; 10: beta-cell dysfunction; 10: obesity-insulin resistance), which were highly associated with Wnt, and G-protein signaling. The anti-diabetic drug metformin activated signaling pathways upstream; whereas pioglitazone inhibited pathways downstream of PAK. Overall, clinical response upon pioglitazone treatment was 53%. Seventy-nine percent of pioglitazone and 75% of metformin treated patients had adverse events. Pioglitazone reduced molecular-PAK biomarkers of proliferation (Ki67 and CyclinD1), and metformin had the opposite effect. **Conclusions**: PAK signaling in T2DM likely involves Wnt and G-protein signaling, which may be altered by the anti-diabetic drugs metformin and pioglitazone. Apart from the therapeutic limitations of adverse events, pioglitazone may be promising in chemoprevention. However long-term multi-centered studies, which initiate pioglitazone treatment early will be required to fully assess the full potential of these drugs.

## 1. Introduction

Type 2-diabetes mellitus (T2DM) is a global epidemic that significantly reduces the quality of life of geriatric patients, especially in Western society. The etiology of T2DM is intimately linked to obesity, genetics, and a sedentary lifestyle. Disease manifestations, including blindness and neuropathy, are extremely burdensome, and long-term complications such as cardiovascular disease and renal failure, ultimately result in death [1].

Although the pathogenesis of T2DM is multifactorial and complex, its current understanding encompasses hepatic insulin resistance, dysfunctional insulin signaling, abnormal glucose metabolism, and persistent hyperglycemia [1]. In addition to disease specific complications, several consequences of hyperglycemia have been described, such as an aberrant immune response, chronic inflammation, and tumorigenesis [2]. Cancer is increasingly common in the elderly, but persistent diabetes also increases the lifelong risk of developing pancreatic, liver, and colorectal cancers and also fuels the tumor microenvironment in cancer patients [2].

Anti-diabetic drugs such as biguanides, sulfonylureas, and glitazones have provided the current basis of understanding in the clinical management of T2DM. However, little is known regarding whether these drugs are also effective in reducing the associated cancer risk. Effective chemoprevention in T2DM will target processes involved in both glucose metabolism and carcinogenesis.

Molecular targets with emerging roles in both cancer and diabetes include a family of six different kinases, the p-21 activated kinases (PAKs) [3]. Here, we reviewed the literature to further understand PAK signaling in T2DM. We aimed to correlate the potential effects of anti-diabetic drugs on PAK signaling in order to provide insight on utilizing PAK signaling as a molecular target in cancer chemoprevention in diabetic patients.

## 2. Methods

### 2.1. Molecular Analysis of PAK Signaling Pathways and Their Involvement in Response to Anti-Diabetic Drugs

A systematic literature review and molecular analysis of pre-clinical studies using online library Pubmed (https://www.ncbi.nlm.nih.gov/pubmed) was performed to establish upstream and downstream PAK targets. Key words included PAK signaling or p-21 activated kinases in addition to the following targets: AMPK, RAS, mTOR, PI3K/AKT, RAC1, CDC42, MAPK, p38, JNK, NF-κB, PPARγ, ROS, VEGF, and Wnt/Beta-catenin. Similar studies were screened for PAK signaling pathways involving known targets (above) of glucose homeostasis, inflammation, proliferation, survival, and angiogenesis. Both upstream and downstream PAK targets were identified in each of these pathways, and the effect of PAK signaling targets on inflammation, proliferation, survival, and angiogenesis was evaluated. Studies in the literature involving anti-diabetics drugs (glitazones, metformin, glyburide) were further analyzed for their effect on PAK signaling pathways (inflammation, proliferation, survival, angiogenesis) on targets upstream and downstream of PAKs.

### 2.2. Identification of PAK Interacting Partners

PAK interacting partners were identified using the STRING database as seen in [4,5]. Confidence was set to 0.40 and active prediction methods, neighborhood, gene fusion, co-occurrence, co-expression, experiments, database, and text mining analysis were performed. Interacting partners were identified in three different conditions which may predispose to T2DM using targets of reduced beta-cell mass (CDKAL1, CDKN2A, CDKN2B), beta cell dysfunction (MTNR1B, TCF7L2, KCNJ11), and obesity/insulin resistance (FTO, IRS1, PPARγ), as reported [1]. Novel PAK interacting partners were further investigated and their pathophysiological role in T2DM was investigated and references for further exploration of targets were provided. All PAK partners were analyzed according to the molecular pathway involved in T2DM.

### 2.3. Clinical Study Inclusion/Exclusion Criteria and Mini-Meta-Analysis

Human clinical studies were identified on ClinicalTrial.gov. Inclusion criteria consisted of observational or interventional studies using metformin or pioglitazone in patients with diseases previously identified in the literature to have increased PAK expression levels such as bladder, leukoplakia, lung, prostate, esophageal, and colorectal cancer [3]. Studies with molecular biomarker analysis or targets downstream of PAKs were included. Studies without data were excluded from analysis. Clinical analyses of overall response rate, adverse events, as well as molecular analysis of biomarkers were performed on pooled data from pioglitazone or metformin treated patients. Observations were analyzed using statistical analysis of variance (ANOVA) and Tukey HSD post hoc tests set at 95% confidence intervals. Calculations were performed using the online tool located at http://statpages.info/anova1sm.html. Chi-squared and independent sample Student’s *t*-tests were performed using the online calculator located at www.socscistatistics.com. Significant *p* values were <0.05.

## 3. Results

### 3.1. PAK Signaling Is Associated with Diabetes and Cancer

Previous studies have provided a role for PAK in both diabetes and cancer, however a clear overview of the signaling pathways involved in both diseases has not been performed. Systematic review of the literature correlated 14 signaling pathways, which were identified as crucial to PAK, either upstream or downstream of PAK, and 11 or 78% of these pathways were also associated with glucose homeostasis (Table 1 and Table 2; Supplementary Figures S1 and S2). All of the PAK signaling pathways which were associated with glucose homeostasis were also associated with malignant inflammatory, proliferative, survival, and angiogenic signaling, which occurs in diseases such as cancer (Table 3 and Supplementary Figure S3).

### 3.2. PAK Interacting Partners Are Associated with the Pathogenesis of T2DM

**Figure 1:**
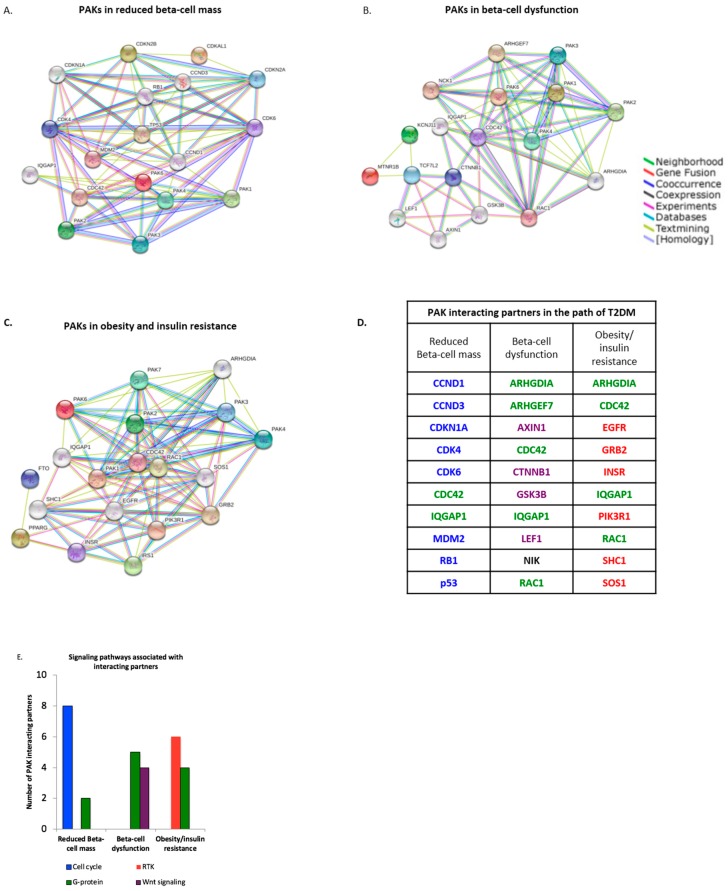
(**A**–**E**) PAK interacting partners are associated with pathogenesis of T2DM. PAK interacting partners were identified with STRING database, using known targets involved in (**A**) reduced beta-cell mass, (**B**) beta-cell dysfunction, and (**C**) obesity/insulin resistance. (**D**) Novel PAK interacting partners involved in reduced beta-cell mass, beta cell dysfunction, and obesity/insulin resistance are displayed and color coded based on their involvement in the cell cycle (blue); G-proteins (green); receptor tyrosine kinase (red); and Wnt signaling (purple). (**E**) Bar graphs indicate the number of interacting partners involved in pathway associated with pathogenesis of T2DM. Reduced beta cell mass is associated with the cell cycle, beta cell dysfunction is associated with Wnt signaling, and obesity/insulin resistance is associated with receptor tyrosine kinases. G-proteins are associated with all three pathways leading to T2DM.

The pathogenesis of T2DM was previously linked to obesity, insulin resistance, and molecular alterations of pancreatic beta cells [1]. PAK signaling was correlated to pathways involved in glucose homeostasis (Table 2); but whether PAK or its partners are associated in the pathogenesis of T2DM is unknown. We identified protein-protein interactions and prospective interacting partners in the pathogenesis of T2DM by screening PAKs 1–6 and previously identified markers of reduced beta cell mass (Figure 1A), dysfunction (Figure 1B), and obesity and insulin resistance (Figure 1C), using the online STRING database [4]. 

Thirty total PAK interacting partners were found to involve reduced Beta-cell mass (10), Beta-cell dysfunction (10), and obesity and insulin resistance (10) (Figure 1D and Supplementary Table S1). Signaling pathways associated with PAK interacting partners included cell cycle control, receptor tyrosine kinases (RTK), G-proteins, and Wnt signaling (Figure 1E). Reduction in Beta-cell mass was exclusively linked to eight-interacting partners involving cell cycle control. Beta-cell dysfunction was found to be associated with four-interacting partners linked to Wnt signaling, and six-interacting partners involving RTKs were associated with obesity/insulin resistance (Figure 1E). G-protein signaling involved two-interacting partners involved in reduced Beta-cell mass, five-partners in Beta-cell dysfunction, and four-partners in obesity/insulin resistance (Figure 1E). These data suggest PAK interacting partners are associated with, and potentially utilize, the cell cycle, Wnt, RTK, and G-protein signaling in the pathogenesis of T2DM.

### 3.3. Upstream and Downstream PAK Signaling Pathways Are Utilized by Anti-Diabetic Drugs Pioglitazone and Metformin

**Figure 2:**
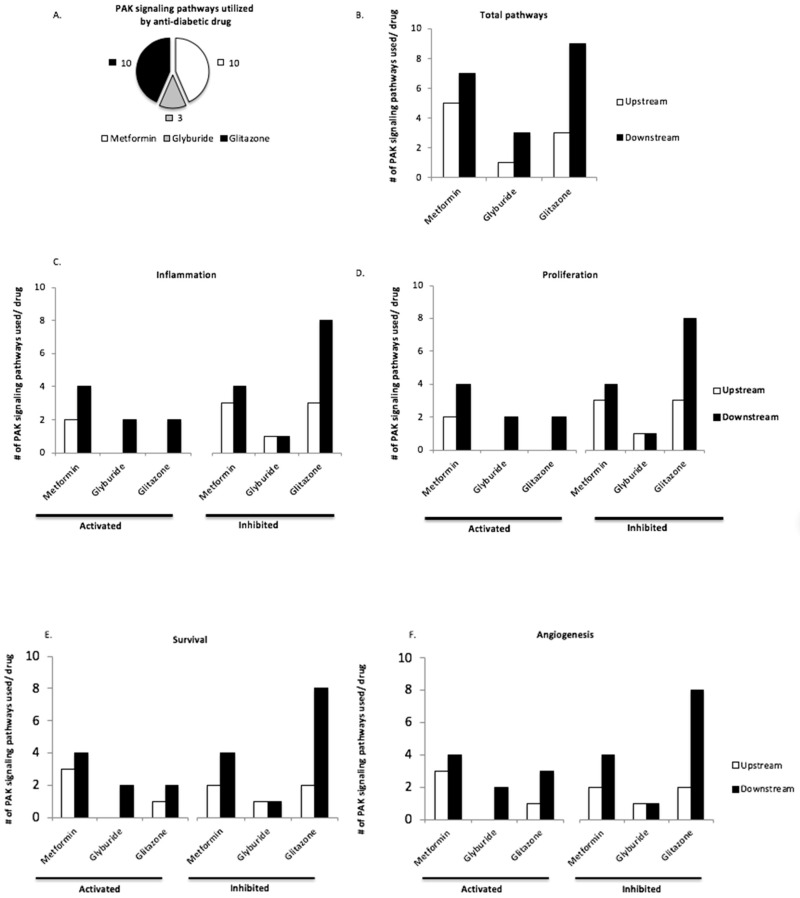
(**A**–**F**) Anti-diabetic drugs utilize upstream and downstream PAK signaling pathways. (**A**) Pie graph indicates total number of PAK signaling pathways utilized by metformin, glyburide, or glitazone. (**B**) Total number of pathways utilized by anti-diabetic drugs upstream or downstream of PAK. Note that total number of pathways in (**A**,**B**) are not equal as pathways interfered by drug may involve targets both up and downstream PAK. All bar graphs indicate the number of PAK signaling pathways involved in inflammation (**C**), proliferation (**D**), survival (**E**), and angiogenesis (**F**) upstream or downstream of PAK, which are either activated or inhibited by anti-diabetic drugs. See text for statistics.

We found that PAK interacting partners were correlated to the pathogenesis of T2DM. However, the role of PAK signaling in T2DM remained elusive. We surveyed this by analyzing the effect of anti-diabetic drugs metformin (met), glyburide (gly), and glitazones (glit) on PAK signaling pathways (Table 4). Overall, glit and met each altered ten PAK signaling pathways, whereas gly altered three (Figure 2A). Further analysis revealed all three anti-diabetic drugs altered pathways upstream and downstream of PAK (Figure 2B). Glit exerted the most profound effect on downstream PAK signaling pathways (mean: glit = 9 vs. met = 7; ANOVA, 95% CI: 0.31 to 3.2, *p* = 0.01) and (mean: glit = 9 vs. gly = 3; ANOVA, 95% CI: 5.3096 to 8.1904, *p* = 0.001). Met altered more pathways upstream of PAK (mean: met = 5 vs. gly = 1; ANOVA, 95% CI = −5.4 to −2.6, *p* = 0.001) and (mean: met = 5 vs. glit = 3; ANOVA, 95% CI = −2.6904 to 0.1904, *p* = 0.11) (Figure 2B).

Malignant PAK signaling is involved in disease pathogenesis. To further understand the role in which met, gly, and glit interfere with PAK signaling in T2DM, we asked if any of these drugs activate or inhibit inflammatory, proliferative, survival, or angiogenic pathways upstream and downstream of PAK (Figure 2C–F). Differential pathway analysis revealed met and glit consistently altered more pathways than gly (Figure 2C–F). Glit inhibited more pathways downstream of PAK than met in (inflammation: glit = 7 vs. met = 4), (proliferation: glit = 8 vs. met = 4), (survival: glit = 8 vs. met = 4), (angiogenesis: glit = 8 vs. met = 4) (Figure 2C–F). However, met activated more pathways than glit, which are exaggerated upstream of PAK and involved in inflammation (met = 4 vs. glit = 2), proliferation (met = 2 vs. glit = 0), survival (met = 3 vs. glit = 1), and angiogenesis (met = 3 vs. glit = 1) (Figure 2C–F). These data suggest that anti-diabetic drugs might alter PAK signaling. Metformin may activate pathways upstream and glitazones potentially inhibit pathways downstream of PAK.

### 3.4. Pioglitazone and Metformin Have Therapeutic Limitations in Cancer Patients with PAK Overexpression

Previous clinical trials have attempted to establish whether met and pioglitazone (pio) therapeutically have chemopreventive activity in humans, but the results remain highly ambiguous. We showed that met and pio altered the pathways both upstream and downstream of PAK, and asked whether the chemopreventive nature of these drugs was found in diseases with PAK overexpression. To investigate this, we analyzed clinical studies in diseases known to overexpress PAK in which met or pio treatment was tested (Table 5).

**Figure 3:**
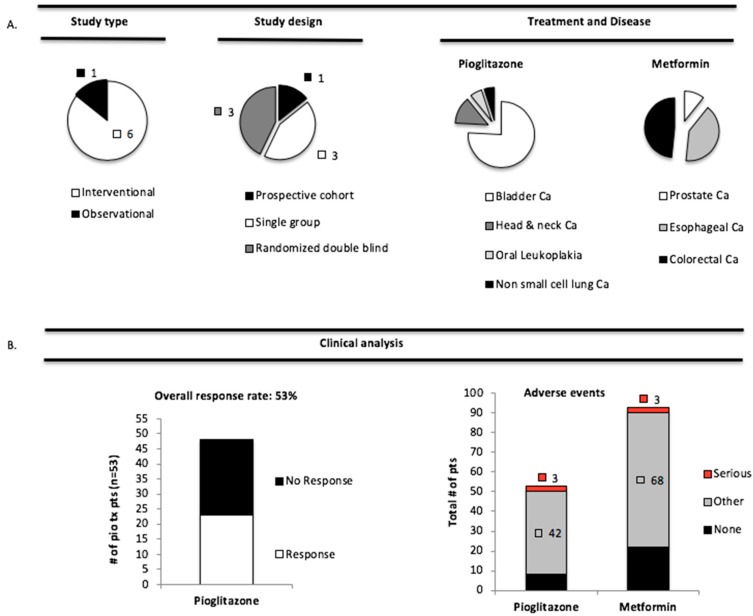
(**A**,**B**) Pioglitazone and metformin have therapeutic limitation in cancer patients. (**A**) Pie graphs demonstrate study type, design, and disease associated with their respective treatment. (**B**) Clinical analyses included calculation of overall response rate and number of serious or other adverse events in pio and met treated patients. Results are pooled data from 53 pio treated patients [NCT00099021 (n = 21), NCT00951379 (n = 26), NCT01342770 (n = 6)] and 91 met treated patients [NCT01433913 (n = 10), NCT01447927 (n = 36), NCT01312467 (n = 45)]. Serious or other adverse events were defined based on ClinicalTrial.gov. See text for statistical analysis.

Initially, we screened hundreds of studies for diseases with PAK overexpression and either met or pio treatment. We found one observational-prospective cohort and six interventional studies (three single arm and three randomized double blind) with these criteria (Table 5 and Figure 3A).

We investigated the effect of anti-diabetic therapy in patients with PAK over-expression by calculating an overall clinical response rate (OCRR), which was defined as the total number of patients who had ≥50% decrease in the sum of all their lesions post-treatment. The OCRR in pio-treated patients was 53% (Figure 3B); 28/53 patients responded and 25/53 did not. The corresponding data for met were unavailable. To further evaluate patient outcome, we calculated the number of serious or other adverse events after pio and met treatment (Figure 3B). Comparison of pio and met treatment revealed a higher percentage of serious (pio: 3/53 = 5.7% vs. met: 3/91 = 3.3% chi-squared, *p* > 0.05) and other (pio: 42/53 = 79% vs. met: 68/91 = 74.7% chi-squared, *p* > 0.05) adverse events upon treatment with pio (Figure 3B). Additionally, in comparison to patients treated with met, a fewer percentage of pio-treated patients were without adverse effects (pio: 11/53 = 21% vs. met: 25/91 = 27.4%) (Figure 3B). Pioglitazone associated events included edema (15%), oral pain (13%), and hypertension (7.5%), while those associated with metformin were gastrointestinal symptoms like constipation (7%), diarrhea (23%), and nausea (13%). These data suggest that patients with tumors, which are known to have PAK overexpression, may have a good clinical response to pio; and both pio and met treatment, are associated with a high rate of mostly mild adverse events.

### 3.5. Pioglitazone and Metformin Alter Biomarkers Downstream of PAK in Human Disease

We correlated how patients which had diseases overexpressing PAK responded to pio, however it was unclear whether pio or met actually interfered with PAK signaling. We therefore analyzed biomarkers downstream of the PAK signaling pathway involved in apoptosis, cell cycle, PI3K/mTOR, and PPARγ (Table 6).

**Figure 4:**
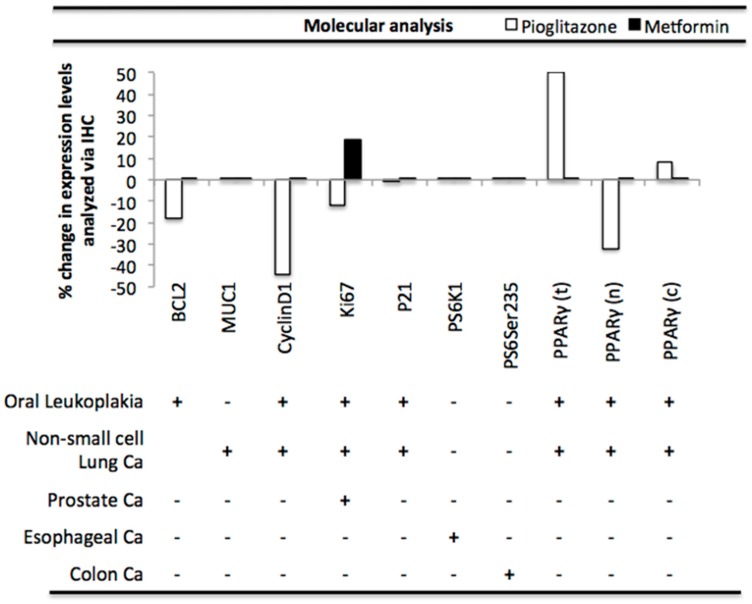
**Pioglitazone and metformin alter biomarkers downstream of PAK in human disease.** Bar graphs are data representing expression of biomarkers calculated by IHC in patients treated with (+ or -) pio [NCT00951379 (n = 25), NCT01342770 (n = 5)] or met [NCT01433913 (n = 8), NCT01447927 (n = 36), NCT01312467 (n = 32)]. See text for statistical analysis.

We calculated the percent change in biomarker expression in pio and met treated patients to visualize the molecular effect on PAK signaling (Figure 4). Molecular analysis revealed that pio treatment decreased expression levels of multiple biomarkers including, apoptotic marker BCL2, and cell cycle markers CyclinD1 and Ki67, respectively. Pio treatment increased total and cytoplasmic PPARγ levels by 50% and 8%, however it decreased nuclear expression by 32% (Figure 4). Met treatment resulted in an increase in cell cycle biomarker Ki67, and did not alter expression levels of PI3K/mTOR markers PS6K1 or PS65Ser235 (Figure 4). Further analysis showed pio inhibited biomarkers involved in cell survival [mean: −12 ± 15; n = 6], whereas met stimulated similar pathways [mean: 12.5 ± 10.6; n = 3] (*p* = 0.042; independent samples *t*-test).

## 4. Discussion

**Figure 5:**
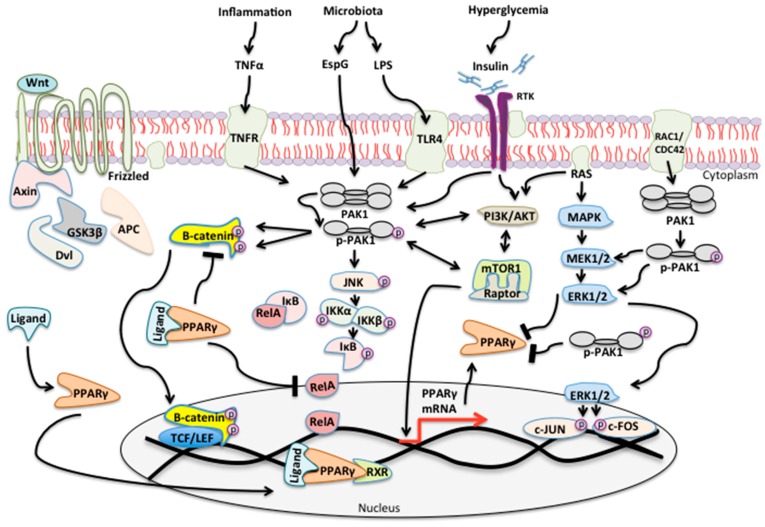
Role of PAK signaling in response to inflammation, altered microbiota and hyperglycemia. Image is read from left to right. (**Far left**) Binding of a Wnt ligand to the Frizzled receptor disrupts the multi-subunit destruction complex consisting of Axin, Dvl, GSK3beta, and APC, which normally tags Beta-catenin to the proteasome. Here, beta-catenin is no longer degraded but moves to the nucleus where it binds to TCF/LEF and initiates transcription of target genes involved in cell cycle progression, proliferation and survival. (**Low left**) Ligand binding to PPARγ results in its activation and nuclear translocation where it then binds RXR and induces its own transcription as well as genes involved in glucose homeostasis and lipid transport. PPARγ directly inhibits Beta-catenin and the NF-κB subunit RelA. (**Central**) Inflammatory cytokines like TNFalpha or microbial products such as EspG or lipopolysaccharides (LPS), result in PAK1 activation. PAK1 phosphorylates Beta-catenin and leads to its stabilization and full transcriptional activation. PAK1 phosphorylates JNK and activates the IKK complex which disrupts RelA from IkB and leading to transcription of genes involved in inflammation and survival. Hyperglycemia results in insulin stimulation of RTKs which further activate PAK1 or directly contribute to a PI3K/AKT/mTOR pathway. PAK1 may also directly contribute to activation of PI3K or mTOR or vice versa. Note that activation of the mTOR1-Raptor complex leads to transcription of PPARγ. As PPARγ levels increase they fulfill their role in the nucleus and cytoplasm. RAS is a key component of the MAPK and PI3K pathway. MAPK-ERK signaling results in activation of JUN and FOS (nucleus) and inhibits PPARγ (cytoplasm). (**Far right**) RAC1/CDC42 are small Rho-GTPases, which also lead to PAK1 activation. PAK1 stimulates the MAPK-ERK cascade and also inhibits PPARγ.

Type 2 diabetes is a major cause of disability and death in the elderly worldwide, and in addition to its multiple disease specific complications, it also carries an increased cancer risk, such as colon cancer [2]. P-21 activated kinases are serine-threonine kinases, which influence multiple cell functions from normal cell signaling to cancer [61]. Physiological PAK signaling is regulated and implicated in the maintenance of cellular homeostasis, however, as the extracellular microenvironment or PAK expression in disease changes, physiological signaling becomes pathological (Figure 5). Here, we reviewed physiological PAK signaling and found it is highly correlated with glucose homeostasis, although PAKs role in the pathogenesis of T2DM, and cancer in diabetes patients, is largely unknown.

PAK overexpression drives malignant diseases such as cancer. However, in diabetes a different expression pattern has been observed by Ahn et al., who reported that diabetic stressors reduce PAK1 expression in islet cells, and this decline in total protein levels may contribute to beta cell dysfunction in diabetes [30]. Here, we emphasized the importance of PAK signaling (kinase activity) versus total protein levels, which are more involved in scaffolding and protein-protein interactions [3]. It is likely that chronic inflammation and hyperinsulinemia associated with T2DM [2,62,63] may both lead to pancreatic and peripheral PAK kinase activation, in addition to reduction in total PAK protein levels, as PAK1 activation was previously described to down regulate itself after activation [64]. Additionally, six different PAKs have been characterized; therefore signaling from other PAKs may compensate for reduction in PAK1 expression, which may have also contributed to the findings observed by Ahn and colleagues. We identified novel PAK interacting partners associated with the pathogenesis of T2DM, involving reduced beta cell mass and dysfunction, and obesity-insulin resistance (Figure 1). Interestingly, we identified that the PAK interacting partner IQGAP1 was involved in all three pathways (Figure 1D). IQGAP1 acts as a molecular scaffold for small Rho-GTPase activation of PAKs [65], which further implicates the importance of PAK kinase activation in T2DM.

The initial pathogenesis of T2DM involves beta cell expansion to compensate for hyperglycemia, which eventually may lead to reduced beta cell mass [1]. Here, PAK interacting partners involved with reduced beta cell mass and dysfunction were specifically associated with cell cycle regulation and Wnt signaling (Figure 1D). It is possible that in early diabetes, hyperglycemia promotes cell cycle progression via PAK-beta-catenin signaling [19]. Over time, PAK driven proliferation and inflammation may lead to oxidative stress and therefore contribute to beta cell dysfunction and subsequent reduction in beta cell mass [66]. Interestingly, in oxidative stress, nuclear beta-catenin was reported to associate with FOXO transcription factors [67], which could potentially modulate PAK expression levels [68], in line with reports from Ahn and colleagues. Another explanation for PAK’s role here may involve an interaction with tumor suppressor p53 (Figure 1A,D). Interestingly, activation of both p53 and MDM2, a p53 ubiquitin ligase, was reported in T2DM [69], and a PAK–MDM2 interaction was previously described [70]. Others provided a role for PAK upstream of p53 [71,72], thus PAK signaling in T2DM may activate p53 directly, or indirectly via MDM2, or through a cell stress pathway, such as oxidative stress or stress associated MAPK like p38/JNK [73], thereby inducing apoptosis, impeding cell cycle progression, and subsequently reducing beta cell mass. Additional PAK interacting partners associated with pathways involving obesity-insulin resistance involved targets in RTK signaling, which is likely a consequence of multiple growth factors, and the chronic inflammatory state associated with obesity in T2DM [1,3,62,63].

We sought to illuminate the role of PAK signaling in T2DM by analyzing the effect that three well-known classes of anti-diabetic drugs had on PAK signaling (Figure 2). Biguanide (metformin), sulfonylurea (glyburide), and glitazone (pioglitazone) all interfered with signaling upstream and downstream of PAKs, and this effect was more significant with metformin (upstream) and pioglitazone (downstream, Figure 2). Both metformin and pioglitazone mediated inflammatory, proliferative, survival, and angiogenic pathways associated with PAKs (Table 4 and Figure 2). However, it is important to note that even though PAK signaling appears to be modulated by these drugs, one major limitation of our study is a lack of an experimental model to support this, therefore we can only speculate using the evidence we found in the literature.

Considering the role of PAK signaling in the initiation of disease [61], and that activation of PAK signaling was correlated to T2DM (Table 2), and potentially inhibited anti-diabetic drugs (Figure 2), we asked whether metformin or pioglitazone were beneficial in patients with PAK-overexpressing diseases including oral cancer [74], non-small cell lung cancer [75], prostate [70], esophageal cancer [76], bladder [77], and colorectal cancer [78]. Pre-clinical studies investigating the chemopreventive effects of these drugs seemed promising [79,80,81], however, results from human studies remain highly ambiguous [82], and a more complete analysis of this data would allocate whether inhibition of PAK signaling pathways by metformin or pioglitazone is promising for chemoprevention. We utilized a mini-meta-analysis of several studies (Table 5) involving metformin and pioglitazone use in cancer patients to investigate whether these treatments are potential candidates to target PAK signaling (Figure 3). However, it is important to note our findings here are only speculation and we cannot definitively conclude that PAK is truly altered by met or pio in these reviewed studies. Also, modulation of PAK signaling by these agents is largely dependent on a particular cell type and tissue context, which is lost when combining studies with different diseases. Nonetheless, our analysis was still able to link that pioglitazone treated patients had a clinical response rate of 53% in PAK dependent diseases (Figure 3B). It was unfortunate that data needed to calculate response rate to metformin were not available for analysis. Both pioglitazone and metformin treatment resulted is relatively few serious adverse events. However, the overwhelming majority of patients, nearly 80% of pioglitazone and 75% metformin treatment patients had mild adverse events (Figure 3B). The high number of pioglitazone associated events such as edema and hypertension were likely due to the advanced treatment regimen used in these patients, equivalent to 45 mg/day, versus the standard care at a dose equivalent to 15 mg/day [83]. Other studies have indicated fewer adverse events at lower doses, equivalent to 7.5 mg/day [83]. Although gastrointestinal symptoms like constipation, diarrhea, and nausea are common side effects of metformin [63], so many adverse events were unexpected and likely not attributable to dose, as patients received a standard of care equivalent of 2000 mg/day. Given that metformin is the gold standard in treatment of T2DM [63], the frequency of adverse events is concerning. Although not life threatening, adverse events are a serious therapeutic limitation and concern for future chemopreventive studies, as long-term patient compliance will dramatically decline if quality of life is decreased by therapy. In addition to our analysis, ten-year treatment of pioglitazone in diabetic patients was associated with increased prostate and pancreatic cancer risk [82], making its long-term use in chemoprevention questionable.

We asked whether PAK signaling was even affected in these patients by pioglitazone or metformin at the molecular level (Table 6 and Figure 4), and found that pioglitazone but not metformin decreased PAK signaling pathways. This data was in line with our analysis of signaling pathways affected by these drugs (Table 4), in that metformin induced signaling upstream of PAKs while pioglitazone rather reduced downstream signaling (Figure 2). Here pioglitazone reduced markers of proliferation and survival, including cyclinD1 and Ki67, while metformin had the opposite effect. Long-term treatment with metformin in diabetic patients may therefore induce, not inhibit, long-term cancer risk, however future studies would need to investigate this further. However, the recent work of Bradley and others show metformin may have long-term chemopreventive effects in preventing colorectal cancer in male diabetic patients [84]. Due to our small sample size in this study we were unable to divide or based on gender or colon cancer, which may have masked similar findings. Also, recent reports from other groups have shown that metformin exerts chemopreventive effects via stimulation of an AMPK-TET-2 tumor suppressor pathway [85], which to our knowledge is separate from PAK signaling pathways discussed in this review.

Considering our clinical and molecular analysis of PAK biomarkers in pioglitazone treated patients, future studies should investigate the long term side effects associated with its treatment and whether PAK signaling in disease can be impeded early on, to block malignant transformation [61]. Although PAK signaling appears to promote malignant disease, whether inhibition of PAK will exacerbate diabetes in these patients is of serious concern. Each case will ultimately need to be analyzed on a risk-reward basis, and patients with both diabetes and increased cancer risk such as (BRCA/Lynch Syndrome/FAP) would be more favorable candidates for long-term studies with these potentially chemopreventive agents.

This was the first study to provide a mechanistic explanation of how anti-diabetics may target PAK signaling for their potential use for chemoprevention in patients with T2DM, albeit our signaling pathway analysis included multiple pre-clinical studies, which were not of high significance. Ideally, a thorough signaling pathway analysis should use PAK1 and p-PAK1 as biomarkers in multiple human studies, however this data was unavailable for analysis due to the limitations of the current literature in regard to our highly specific question. Considering the few patients analyzed here, the accuracy of this data is supported by its correlation between literature reports (Table 4 and Figure 2) and our analysis of PAK signaling pathways from human studies (Table 6 and Figure 4). Future studies with more patients and additional readouts of known PAK targets would provide a more clear analysis of whether chemoprevention in diabetes with pioglitazone is feasible long term.

### 4.1. Future Directions

Although not associated with diabetes, other studies of chemoprevention have shown that anti-inflammatory drugs like aspirin in colorectal cancer and mesalamine in colitis associated cancer reduce cancer-associated risk [86,87]. Mesalamine, the first line treatment for chronic inflammation in ulcerative colitis [88], was recently established as a PAK1 inhibitor [17], and others have shown it is a PPARγ ligand [89]. Both of these mechanisms are in line with glitazones [14]. Therefore, future directions in chemoprevention in diabetes should analyze PAK expression/phosphorylation upon glitazone treatment and see if the effects are similar to those of mesalamine in impeding chronic inflammation in colitis associated colon cancer.

### 4.2. Concluding Remarks

Anti-diabetics like pioglitazone or metformin should be utilized as a platform for further understanding the role of PAKs as a chemopreventive target in diabetes. However, before this is possible, future studies must standardize doses specific to the associated disease in order to modulate PAK signaling appropriately and minimize adverse effects. Ideal chemoprevention, like mesalamine, will block inflammation and impede aberrant PAK signaling without altering cellular homeostasis.

## Figures and Tables

**Table 1:** Upstream and downstream PAK signaling pathways. Upstream (left) pathways result in PAK activation. Activated PAK contributes to multiple downstream signaling pathways (right).

Upstream		Downstream	
AMPK	[6]	MAPK-p38/JNK	[7]
RAS	[8]	MAPK-ERK	[9]
mTOR	[10,11]	mTOR	[12]
PI3k/AKT	[13]	PI3k/AKT	[9,12,14]
RAC1	[15]	NF-kB	[14]
CDC42	[15]	p-PAK1	[14]
		PPARy	[14]
		ROS	[16]
		VEGF	[9]
		Wnt/B-catenin	[17,18,19]

**Table 2:** PAK signaling pathways are involved in glucose homeostasis. The role of PAK in glucose homeostasis is complex. Each PAK signaling pathway plays a unique part in glucose homeostasis, and significant overlap exists between each of the pathways listed.

PAK Signaling Pathways in Glucose Homeostasis	
	Reference
AMPK	[6,20]
MAPK-p38/JNK	[21]
MAPK-ERK	[22]
mTOR	[23]
NF-kB	[24]
p-PAK1	[25]
PI3K/AKT	[26]
PPARy	[27]
RAC1/CDC42	[28,29]
RAS	[30]
Wnt/Beta-catenin	[31]

**Table 3:** Role of PAK signaling in disease. PAK activation or overexpression in malignant disease results in activation of multiple signaling pathways, which drive inflammation, proliferation, survival, and angiogenesis. Arrows indicate the effect of signaling pathways on inflammation, proliferation, survival, and angiogenesis.

Role of PAK Signaling Pathways in Disease					
Pathway	Inflammation	Proliferation	Survival	Angiogenesis	Reference
AMPK	↓	↓	↑	↑	[32,33,34,35]
MAPK-p38/JNK	↑	↑	↑	↑	[36]
MAPK-ERK	↑	↑	↑	↑	[9]
mTOR	↑	↑	↑	↑	[37]
NF-kB	↑	↑	↑	↑	[14]
p-PAK1	↑	↑	↑	↑	[38]
PI3k/AKT	↑	↑	↑	↑	[37]
PPARy	↓	↓	↓	↑↓	[39,40]
RAC1/CDC42	↑	↑	↑	↑	[15,18,26,28]
ROS	↑	↑↓	↑↓	↑↓	[16,41]
VEGF	↑	↑	↑	↑	[42,43]
Wnt/B-catenin	↑	↑	↑	↑	[17,18,19,38]

**Table 4:** PAK-signaling pathways are utilized by anti-diabetic drugs. The effect of anti-diabetic drugs metformin, glyburide, and pioglitazone on PAK signaling pathways as reported by the literature. Arrows indicate the effect of drug on PAK signaling pathway up (increases); down (decreases); up/down (both).

PAK Signaling Pathways Utilized by Anti-diabetic Drugs				
Pathway	Metformin	Glyburide	Glitazone	Citation
AMPK	↑	-	↑	[44,45]
MAPK-p38/JNK	↑	↑	↓	[46,47,48,49]
MAPK-ERK	↓	-	↑↓	[47,50]
mTOR	↓	-	↓	[51,52,53]
NF-kB	↓	-	↓	[14,38,54,55]
p-PAK1	↑	-	-	[44]
PI3K/AKT	↑	↓	↓	[37,48]
PPAR-γ	-	-	↑	[27]
RAC1/CDC42	↑	-	-	[47]
RAS	↓	-	-	[51]
ROS	-	↑	↓	[56]
VEGF	↑↓	-	↑↓	[57,58]
Wnt/ B-catenin	-	-	↓	[59,60]

**Table 5:** Clinical studies using pioglitazone or metformin in diseases with PAK overexpression. Overview of clinical studies analyzed for their effect of anti-diabetic drug pioglitazone (pio) or metformin (met) on PAK signaling pathways in diseases known to overexpress PAK.

Summary of Clinical Study Designs							
Study Type	Design	Condition	Intervention	Primary Outcome Measure	Number of Patients Treatment (tx); Control (con)	Duration	Identifier
Observational	Prospective cohort	Diabetes Bladder Ca	Pioglitazone	Incident Diagnoses	Tx: 34,181 Con: 158,918	10 years	NCT01637935
Interventional	Single group; prevention	Head/Neck Ca; Oral Leukoplakia	Pioglitazone	Overall response	Tx: 21 Con: 0	12 weeks	NCT00099021
Interventional	Randomized; double blind; treatment	Oral Leukoplakia	Pioglitazone	Overall response	Tx: 27 Con: 25	24 weeks	NCT00951379
Interventional	Single group; Treatment	Non Small cell lung Ca	Pioglitazone	% change Ki67 IHC	Tx: 6 Con: 0	14–42 days	NCT01342770
Interventional	Randomized; double blind; treatment	Prostate adenocarcinoma	Metformin	% change Ki67 IHC	Tx: 10 Con: 10	4–12 weeks	NCT01433913
Interventional	Randomized; double blind; prevention	Barrett Esophagus; Esophageal Ca	Metformin	% change pS6K1 IHC	Tx: 38 Con: 36	3 months	NCT01447927
Interventional	Single group; prevention	Adenomatous polyp; CRC; obesity	Metformin	% change S6-serine235	Tx: 45 Con: 0	12 weeks	NCT01312467

**Table 6:** Pioglitazone and metformin alter biomarkers downstream of PAK in human disease. Clinical trials, which utilized pio or met in diseases with PAK overexpression, were analyzed for their immunohistochemistry data. Biomarkers: apoptosis (BCL2, MUC1), cell cycle control (CyclinD1, Ki67, P21), PI3K/mTOR (PS6K1, PS6Ser235), and PPARγ. Symbols indicate (+) treatment or (-) not available. (up or down arrow; 0) indicates percent increase or decrease in expression analyzed via IHC. 0 (no change); 1 arrow (>1% change): 2 arrows (>10% change); 3 arrows (>20% change); 4 arrows (>50% change).

Biomarker Analysis in Cancer Patients Treated with Pioglitazone or Metformin													
Disease	Drug		Marker analyzed via immunohistochemistry (IHC)										Study ID
			Apoptosis		Cell cycle control			PI3K/mTOR		PPARy			
	pioglitazone	metformin	BCL2	MUC1	CyclinD1	Ki67	P21	PS6K1	PS6ser235	Total	Nuclear	Cytoplasm	
Oral leukoplakia	**+**	-	↓ ↓	-	↓ ↓	↓	↑	-	-	-	↓ ↓ ↓	↑	NCT0095 1379
Non-small cell lung Ca	**+**	-	-	**0**	↓ ↓ ↓ ↓	↓ ↓	↓	-	-	↑ ↑ ↑ ↑	-	-	NCT0134 2770
Prostate Ca	-	**+**	-	-	-	↑ ↑	-	-	-	-	-	-	NCT0143 3913
Esophageal Ca	-	**+**	-	-	-	-	-	↑	-	-	-	-	NCT0144 7927
Colon Ca	-	**+**	-	-	-	-	-	-	**0**	-	-	-	NCT0131 2467

## Supplementary Materials

The following are available online, Figure S1: Role of signaling pathways upstream and downstream of p-21 activated kinases. Figure S2: Diverse roles of PAKs in glucose homeostasis. Figure S3: PAK signaling in disease. Table S1: The pathophysiology of PAK interacting partners in T2DM.

## References

[1] McCarthy M.I. (2010). Genomics, type 2 diabetes, and obesity. N. Engl. J. Med..

[2] Chang S.-C., Yang W.-C.V. (2016). Hyperglycemia, tumorigenesis, and chronic inflammation. Crit. Rev. Oncol./Hematol..

[3] Dammann K., Khare V., Gasche C. (2014). Tracing PAKs from GI inflammation to cancer. Gut.

[4] Szklarczyk D., Franceschini A., Wyder S., Forslund K., Heller D., Huerta-Cepas J., Simonovic M., Roth A., Santos A., Tsafou K.P. (2015). STRING v10: Protein-protein interaction networks, integrated over the tree of life. Nucleic Acids Res..

[5] Campregher C., Schmid G., Ferk F., Knasmuller S., Khare V., Kortum B., Dammann K., Lang M., Scharl T., Spittler A. (2012). MSH3-deficiency initiates EMAST without oncogenic transformation of human colon epithelial cells. PLoS ONE.

[6] Kong D., Dagon Y., Campbell J.N., Guo Y., Yang Z., Yi X., Aryal P., Wellenstein K., Kahn B.B., Sabatini B.L. (2016). A Postsynaptic AMPK-->p21-Activated Kinase Pathway Drives Fasting-Induced Synaptic Plasticity in AgRP Neurons. Neuron.

[7] Dodeller F., Schulze-Koops H. (2006). The p38 mitogen-activated protein kinase signaling cascade in CD4 T cells. Arthritis Res. Ther..

[8] Menard R.E., Mattingly R.R. (2003). Cell surface receptors activate p21-activated kinase 1 via multiple Ras and PI3-kinase-dependent pathways. Cell. Signal..

[9] Huynh N., Liu K.H., Baldwin G.S., He H. (2010). P21-activated kinase 1 stimulates colon cancer cell growth and migration/invasion via ERK- and AKT-dependent pathways. Biochim. Biophys. Acta.

[10] Gu S., Kounenidakis M., Schmidt E.-M., Deshpande D., Alkahtani S., Alarifi S., Föller M., Alevizopoulos K., Lang F., Stournaras C. (2013). Rapid activation of FAK/mTOR/p70S6K/PAK1-signaling controls the early testosterone-induced actin reorganization in colon cancer cells. Cell. Signal..

[11] Ishida H., Li K., Yi M., Lemon S.M. (2007). p21-activated kinase 1 is activated through the mammalian target of rapamycin/p70 S6 kinase pathway and regulates the replication of hepatitis C virus in human hepatoma cells. J. Biol. Chem..

[12] Khare V., Dammann K., Asboth M., Krnjic A., Jambrich M., Gasche C. (2015). Overexpression of PAK1 promotes cell survival in inflammatory bowel diseases and colitis-associated cancer. Inflamm. Bowel Dis..

[13] Ijuin T., Takenawa T. (2012). Regulation of Insulin Signaling by the Phosphatidylinositol 3,4,5-Triphosphate Phosphatase SKIP through the Scaffolding Function of Pak1. Mol. Cell. Biol..

[14] Dammann K., Khare V., Lang M., Claudel T., Harpain F., Granofszky N., Evstatiev R., Williams J.M., Pritchard D.M., Watson A. (2015). PAK1 modulates a PPARγ/NF-κB cascade in intestinal inflammation. Biochim. Biophys. Acta (BBA) Mol. Cell Res..

[15] Shin Y.J., Kim E.H., Roy A., Kim J.-H. (2013). Evidence for a novel mechanism of the PAK1 interaction with the Rho-GTPases Cdc42 and Rac. PLoS ONE.

[16] DeSantiago J., Bare D.J., Xiao L., Ke Y., Solaro R.J., Banach K. (2014). p21-Activated kinase1 (Pak1) is a negative regulator of NADPH-oxidase 2 in ventricular myocytes. J. Mol. Cell. Cardiol..

[17] Khare V., Lyakhovich A., Dammann K., Lang M., Borgmann M., Tichy B., Pospisilova S., Luciani G., Campregher C., Evstatiev R. (2013). Mesalamine modulates intercellular adhesion through inhibition of p-21 activated kinase-1. Biochem. Pharmacol..

[18] Zhu G., Wang Y., Huang B., Liang J., Ding Y., Xu A., Wu W. (2012). A Rac1/PAK1 cascade controls beta-catenin activation in colon cancer cells. Oncogene.

[19] Park M.-H., Kim D.-J., You S.-T., Lee C.-S., Kim H.K., Park S.M., Shin E.Y., Kim E.G. (2012). Phosphorylation of beta-catenin at serine 663 regulates its transcriptional activity. Biochem. Biophys. Res. Commun..

[20] Viollet B., Lantier L., Devin-Leclerc J., Hébrard S., Amouyal C., Mounier R., Foretz M., Andreelli F. (2009). Targeting the AMPK pathway for the treatment of Type 2 diabetes. Front. Biosci..

[21] Sozen B., Ozturk S., Yaba A., Demir N. (2015). The p38 MAPK signalling pathway is required for glucose metabolism, lineage specification and embryo survival during mouse preimplantation development. Mech. Dev..

[22] Zhang W., Thompson B.J., Hietakangas V., Cohen S.M. (2011). MAPK/ERK signaling regulates insulin sensitivity to control glucose metabolism in Drosophila. PLoS Genet..

[23] Altomare D.A., Khaled A.R. (2012). Homeostasis and the Importance for a Balance Between AKT/mTOR Activity and Intracellular Signaling. Curr. Med. Chem..

[24] Mauro C., Leow S.C., Anso E., Rocha S., Thotakura A.K., Tornatore L., Moretti M., De Smaele E., Beg A.A., Tergaonkar V. (2011). NF-kappaB controls energy homeostasis and metabolic adaptation by upregulating mitochondrial respiration. Nat. Cell Biol..

[25] Chiang Y.A., Jin T. (2014). p21-Activated protein kinases and their emerging roles in glucose homeostasis. Am. J. Physiol. Endocrinol. Metab..

[26] Sylow L., Kleinert M., Pehmøller C., Prats C., Chiu T.T., Klip A., Richter E.A., Jensen T.E. (2014). Akt and Rac1 signaling are jointly required for insulin-stimulated glucose uptake in skeletal muscle and downregulated in insulin resistance. Cell. Signal..

[27] Picard F., Auwerx J. (2002). PPAR(gamma) and glucose homeostasis. Annu. Rev. Nutr..

[28] Gautam S., Ishrat N., Singh R., Narender T., Srivastava A.K. (2015). Aegeline from Aegle marmelos stimulates glucose transport via Akt and Rac1 signaling, and contributes to a cytoskeletal rearrangement through PI3K/Rac1. Eur. J. Pharmacol..

[29] Ahn M., Yoder S.M., Wang Z., Oh E., Ramalingam L., Tunduguru R., Thurmond D.C. (2016). The p21-activated kinase (PAK1) is involved in diet-induced beta cell mass expansion and survival in mice and human islets. Diabetologia.

[30] Manchester J., Kong X., Lowry O.H., Lawrence J.C. (1994). Ras signaling in the activation of glucose transport by insulin. Proc. Natl. Acad. Sci. USA.

[31] Elghazi L., Gould A.P., Weiss A.J., Barker D.J., Callaghan J., Opland D., Myers M., Cras-Méneur C., Bernal-Mizrachi E. (2012). Importance of beta-Catenin in glucose and energy homeostasis. Sci. Rep..

[32] Salminen A., Hyttinen J.M.T., Kaarniranta K. (2011). AMP-activated protein kinase inhibits NF-κB signaling and inflammation: Impact on healthspan and lifespan. J. Mol. Med..

[33] Motoshima H., Goldstein B.J., Igata M., Araki E. (2006). AMPK and cell proliferation—AMPK as a therapeutic target for atherosclerosis and cancer. J. Physiol..

[34] Domenech E., Maestre C., Esteban-Martinez L., Partida D., Pascual R., Fernandez-Miranda G., Seco E., Campos-Olivas R., Pérez M., Megias D. (2015). AMPK and PFKFB3 mediate glycolysis and survival in response to mitophagy during mitotic arrest. Nat. Cell Biol..

[35] Ouchi N., Shibata R., Walsh K. (2005). AMP-activated protein kinase signaling stimulates VEGF expression and angiogenesis in skeletal muscle. Circ. Res..

[36] Cuadrado A., Nebreda A.R. (2010). Mechanisms and functions of p38 MAPK signalling. Biochem. J..

[37] Karar J., Maity A. (2011). PI3K/AKT/mTOR Pathway in Angiogenesis. Front. Mol. Neurosci..

[38] Dammann K., Khare V., Harpain F., Lang M., Kurtovic A., Mesteri I., Evstatiev R., Gasche C. (2015). PAK1 promotes intestinal tumor initiation. Cancer Prev. Res..

[39] Salomone S. (2011). Pleiotropic Effects of Glitazones: A Double Edge Sword?. Front. Pharmacol..

[40] Kotlinowski J., Jozkowicz A. (2016). PPAR Gamma and Angiogenesis: Endothelial Cells Perspective. J. Diabetes Res..

[41] Liou G.-Y., Storz P. (2010). Reactive oxygen species in cancer. Free Radic. Res..

[42] Devery A.M., Wadekar R., Bokobza S.M., Weber A.M., Jiang Y., Ryan A.J. (2015). Vascular endothelial growth factor directly stimulates tumour cell proliferation in non-small cell lung cancer. Int. J. Oncol..

[43] Li X., Lee C., Tang Z., Zhang F., Arjunan P., Li Y., Hou X., Kumar A., Dong L. (2009). VEGF-B: A survival, or an angiogenic factor?. Cell Adhes. Migr..

[44] You G.Y., Lee J.O., Kim J.H., Kim N., Lee S.K., Moon J.W., Jie S., Lee H.J., Kim S.J., Park S.H. (2013). Tiam-1, a GEF for Rac1, plays a critical role in metformin-mediated glucose uptake in C2C12 cells. Cell. Signal..

[45] Coletta D.K., Sriwijitkamol A., Wajcberg E., Tantiwong P., Li M., Prentki M., Madiraju M., Jenkinson C.P., Cersosimo E., Musi N. (2009). Pioglitazone stimulates AMP-activated protein kinase signalling and increases the expression of genes involved in adiponectin signalling, mitochondrial function and fat oxidation in human skeletal muscle in vivo: A randomised trial. Diabetologia.

[46] Wu N., Gu C., Gu H., Hu H., Han Y., Li Q. (2011). Metformin induces apoptosis of lung cancer cells through activating JNK/p38 MAPK pathway and GADD153. Neoplasma.

[47] Kumar A., Al-Sammarraie N., DiPette D.J., Singh U.S. (2014). Metformin impairs Rho GTPase signaling to induce apoptosis in neuroblastoma cells and inhibits growth of tumors in the xenograft mouse model of neuroblastoma. Oncotarget.

[48] Qian X., Li J., Ding J., Wang Z., Duan L., Hu G. (2008). Glibenclamide exerts an antitumor activity through reactive oxygen species-c-jun NH2-terminal kinase pathway in human gastric cancer cell line MGC-803. Biochem. Pharmacol..

[49] Okami N., Narasimhan P., Yoshioka H., Sakata H., Kim G.S., Jung J.E., Maier C.M., Chan P.H. (2013). Prevention of JNK phosphorylation as a mechanism for rosiglitazone in neuroprotection after transient cerebral ischemia: Activation of dual specificity phosphatase. J. Cereb. Blood Flow Metab..

[50] Bolden A., Bernard L., Jones D., Akinyeke T., Stewart L.V. (2012). The PPAR gamma agonist troglitazone regulates erk 1/2 phosphorylation via a PPARγ-independent, MEK-dependent pathway in human prostate cancer cells. PPAR Res..

[51] Nair V., Sreevalsan S., Basha R., Abdelrahim M., Abudayyeh A., Hoffman A.R., Safe S. (2014). Mechanism of metformin-dependent inhibition of mammalian target of rapamycin (mTOR) and Ras activity in pancreatic cancer: Role of specificity protein (Sp) transcription factors. J. Biol. Chem..

[52] San Y.-Z., Liu Y., Zhang Y., Shi P.-P., Zhu Y.-L. (2015). Peroxisome proliferator-activated receptor-gamma agonist inhibits the mammalian target of rapamycin signaling pathway and has a protective effect in a rat model of status epilepticus. Mol. Med. Rep..

[53] Blanchard P.-G., Festuccia W.T., Houde V.P., St-Pierre P., Brule S., Turcotte V., Côté M., Bellmann K., Marette A., Deshaies Y. (2012). Major involvement of mTOR in the PPARgamma-induced stimulation of adipose tissue lipid uptake and fat accretion. J. Lipid Res..

[54] Isoda K., Young J.L., Zirlik A., MacFarlane L.A., Tsuboi N., Gerdes N., Schonbeck U., Libby P. (2006). Metformin inhibits proinflammatory responses and nuclear factor-kappaB in human vascular wall cells. Arterioscler. Thromb. Vasc. Biol..

[55] Hou Y., Moreau F., Chadee K. (2012). PPARgamma is an E3 ligase that induces the degradation of NFkappaB/p65. Nat. Commun..

[56] Yuan X., Zhang Z., Gong K., Zhao P., Qin J., Liu N. (2011). Inhibition of reactive oxygen species/extracellular signal-regulated kinases pathway by pioglitazone attenuates advanced glycation end products-induced proliferation of vascular smooth muscle cells in rats. Biol. Pharm. Bull..

[57] Dallaglio K., Bruno A., Cantelmo A.R., Esposito A.I., Ruggiero L., Orecchioni S., Calleri A., Bertolini F., Pfeffer U., Noonan D.M. (2014). Paradoxic effects of metformin on endothelial cells and angiogenesis. Carcinogenesis.

[58] Ersoy C., Kiyici S., Budak F., Oral B., Guclu M., Duran C., Selimoglu H., Erturk E., Tuncel E., Imamoglu S. (2008). The effect of metformin treatment on VEGF and PAI-1 levels in obese type 2 diabetic patients. Diabetes Res. Clin. Pract..

[59] Lu D., Carson D.A. (2010). Repression of beta-catenin signaling by PPAR gamma ligands. Eur. J. Pharmacol..

[60] Wang P.-S., Chou F.-S., Bloomston M., Vonau M.S., Saji M., Espinosa A., Pinzone J.J. (2009). Thiazolidinediones downregulate Wnt/beta-catenin signaling via multiple mechanisms in breast cancer cells. J. Surg. Res..

[61] Kumar R., Li D.-Q. (2016). PAKs in Human Cancer Progression: From Inception to Cancer Therapeutic to Future Oncobiology. Adv. Cancer Res..

[62] Donath M.Y., Shoelson S.E. (2011). Type 2 diabetes as an inflammatory disease. Nat. Rev. Immunol..

[63] Bosi E. (2009). Metformin—The gold standard in type 2 diabetes: What does the evidence tell us?. Diabetes Obes. Metab..

[64] Hubsman M.W., Volinsky N., Manser E., Yablonski D., Aronheim A. (2007). Autophosphorylation-dependent degradation of Pak1, triggered by the Rho-family GTPase, Chp. Biochem. J..

[65] Li R., Debreceni B., Jia B., Gao Y., Tigyi G., Zheng Y. (1999). Localization of the PAK1-, WASP-, and IQGAP1-specifying regions of Cdc42. J. Biol. Chem..

[66] Drews G., Krippeit-Drews P., Dufer M. (2010). Oxidative stress and beta-cell dysfunction. Pflugers Arch. Eur. J. Physiol..

[67] Essers M.A.G., de Vries-Smits L.M.M., Barker N., Polderman P.E., Burgering B.M.T., Korswagen H.C. (2005). Functional interaction between beta-catenin and FOXO in oxidative stress signaling. Science.

[68] Mazumdar A., Kumar R. (2003). Estrogen regulation of Pak1 and FKHR pathways in breast cancer cells. FEBS Lett..

[69] Li X., Cheng K.K.Y., Liu Z., Yang J.-K., Wang B., Jiang X., Zhou Y., Hallenborg P., Hoo R.L., Lam K.S. (2016). The MDM2-p53-pyruvate carboxylase signalling axis couples mitochondrial metabolism to glucose-stimulated insulin secretion in pancreatic beta-cells. Nat. Commun..

[70] Liu T., Li Y., Gu H., Zhu G., Li J., Cao L., Li F. (2013). P21-Activated kinase 6 (PAK6) inhibits prostate cancer growth via phosphorylation of androgen receptor and tumorigenic E3 ligase murine double minute-2 (MDM2). J. Biol. Chem..

[71] Murray B.W., Guo C., Piraino J., Westwick J.K., Zhang C., Lamerdin J., Dagostino E., Knighton D., Loi C.M., Zager M. (2010). Small-molecule p21-activated kinase inhibitor PF-3758309 is a potent inhibitor of oncogenic signaling and tumor growth. Proc. Natl. Acad. Sci. USA.

[72] Park S.-Y., Lee J.H., Ha M., Nam J.-W., Kim V.N. (2009). miR-29 miRNAs activate p53 by targeting p85 alpha and CDC42. Nat. Struct. Mol. Biol..

[73] Shi Y., Nikulenkov F., Zawacka-Pankau J., Li H., Gabdoulline R., Xu J., Eriksson S., Hedström E., Issaeva N., Kel A. (2014). ROS-dependent activation of JNK converts p53 into an efficient inhibitor of oncogenes leading to robust apoptosis. Cell Death Differ..

[74] Parvathy M., Sreeja S., Kumar R., Pillai M.R. (2016). Potential role of p21 Activated Kinase 1 (PAK1) in the invasion and motility of oral cancer cells. BMC Cancer.

[75] Liu Y., Wang S., Dong Q.-Z., Jiang G.-Y., Han Y., Wang L., Wang E.-H. (2016). The P21-activated kinase expression pattern is different in non-small cell lung cancer and affects lung cancer cell sensitivity to epidermal growth factor receptor tyrosine kinase inhibitors. Med. Oncol..

[76] Gan J., Zhang Y., Ke X., Tan C., Ren H., Dong H., Jiang J., Chen S., Zhuang Y., Zhang H. (2015). Dysregulation of PAK1 Is Associated with DNA Damage and Is of Prognostic Importance in Primary Esophageal Small Cell Carcinoma. Int. J. Mol. Sci..

[77] Ito M., Nishiyama H., Kawanishi H., Matsui S., Guilford P., Reeve A., Ogawa O. (2007). P21-activated kinase 1: A new molecular marker for intravesical recurrence after transurethral resection of bladder cancer. J. Urol..

[78] Song B., Wang W., Zheng Y., Yang J., Xu Z. (2015). P21-activated kinase 1 and 4 were associated with colorectal cancer metastasis and infiltration. J. Surg. Res..

[79] Girnun G.D., Smith W.M., Drori S., Sarraf P., Mueller E., Eng C., Nambiar P., Rosenberg D.W., Bronson R.T., Edelmann W. (2002). APC-dependent suppression of colon carcinogenesis by PPARgamma. Proc. Natl. Acad. Sci. USA.

[80] Grommes C., Karlo J.C., Caprariello A., Blankenship D., Dechant A., Landreth G.E. (2013). The PPARgamma agonist pioglitazone crosses the blood-brain barrier and reduces tumor growth in a human xenograft model. Cancer Chemother. Pharmacol..

[81] Burotto M., Szabo E. (2014). PPARγ in Head and Neck Cancer Prevention. Oral Oncol..

[82] Lewis J.D., Habel L.A., Quesenberry C.P., Strom B.L., Peng T., Hedderson M.M., Ehrlich S.F., Mamtani R., Bilker W., Vaughn D.J. (2015). Pioglitazone Use and Risk of Bladder Cancer and Other Common Cancers in Persons With Diabetes. JAMA.

[83] Majima T., Komatsu Y., Doi K., Shigemoto M., Takagi C., Fukao A., Corners J., Nakao K. (2006). Safety and efficacy of low-dose pioglitazone (7.5 mg/day) vs. standard-dose pioglitazone (15 mg/day) in Japanese women with type 2 diabetes mellitus. Endocr. J..

[84] Bradley M.C., Ferrara A., Achacoso N., Ehrlich S.F., Quesenberry C.P.J., Habel L.A. (2018). A Cohort Study of Metformin and Colorectal Cancer Risk among Patients with Diabetes Mellitus. Cancer Epidemiol. Biomark. Prev..

[85] Wu D., Hu D., Chen H., Shi G., Fetahu I.S., Wu F., Rabidou K., Fang R., Tan L., Xu S. (2018). Glucose-regulated phosphorylation of TET2 by AMPK reveals a pathway linking diabetes to cancer. Nature.

[86] Drew D.A., Cao Y., Chan A.T. (2016). Aspirin and colorectal cancer: The promise of precision chemoprevention. Nat. Rev. Cancer.

[87] Lyakhovich A., Gasche C. (2010). Systematic review: Molecular chemoprevention of colorectal malignancy by mesalazine. Aliment. Pharmacol. Ther..

[88] Karagozian R., Burakoff R. (2007). The role of mesalamine in the treatment of ulcerative colitis. Ther. Clin. Risk Manag..

[89] Rousseaux C., Lefebvre B., Dubuquoy L., Lefebvre P., Romano O., Auwerx J., Metzger D., Wahli W., Desvergne B., Naccari G.C. (2005). Intestinal antiinflammatory effect of 5-aminosalicylic acid is dependent on peroxisome proliferator-activated receptor-gamma. J. Exp. Med..

