# Supplementary material for: p-21 Activated Kinase as a Molecular Target for Chemoprevention in Diabetes
Source: Geriatrics (Basel). 2018 Oct 19;3(4):73. doi: 10.3390/geriatrics3040073 (PMC6371191; doi:10.3390/geriatrics3040073)
Supplement: Supplementary file 1 [file geriatrics-03-00073-s001.zip › supplementary/Supp Table 1.docx]

| **Pathophysiological role of interacting partners in T2DM** | | |
| --- | --- | --- |
| **Target** | **Description** | **Reference** |
| ARHGDIA | Rho GDP-dissociation inhibitor 1: RAC1/CDC42 inactivation | (W. Lu et al., 2016) |
| ARHGEF7 | Rho guanine nucleotide exchange factor 7: RAC1 activation | (Nola et al., 2008) |
| AXIN1 | Component of Beta-catenin destruction complex in Wnt signaling | (Clevers, Loh, & Nusse, 2014) |
| CCND1 | G1/S specific cyclin-D1: RB inactivation and promotion of G1/S transition | (Taneera et al., 2013) |
| CCND3 | G1/S specific cyclin-D3: RB inactivation and promotion of G1/S transition | (Marselli et al., 2010) |
| CDKN1A | Cyclin dependent kinase inhibitor 1: impedes cell cycle progression | (Taneera et al., 2013) |
| CDK4 | G1/S specific cyclin dependent kinase 4: RB inactivation and promotion of G1/S transition | (Taneera et al., 2013) |
| CDK6 | G1/S specific cyclin dependent kinase 6: RB inactivation and promotion of G1/S transition | (Taneera et al., 2013) |
| CDC42 | Cell division control protein 42: GTPase which activates PAKs | (Raut et al., 2015) |
| CTNNB1 | Component of Wnt signaling and adherent junctions at cell membrane | (Clevers et al., 2014) |

| EGFR | Epidermal growth factor receptor: receptor tyrosine kinase activates RAS, MAPK, PAK1, PI3 kinase | (Tomar & Schlaepfer, 2010) |
| --- | --- | --- |
| GRB2 | Growth factor receptor bound protein 2: adaptor protein linking EGFR to RAS | (Puto, Pestonjamasp, King, & Bokoch, 2003) |
| GSK3B | Component of Beta-catenin destruction complex | (Clevers et al., 2014) |
| INSR | Insulin receptor: receptor tyrosine kinase activates MAPK and PI3K | (Yuting Alex Chiang, Shao, Xu, Chernoff, & Jin, 2013) |
| IQGAP1 | RAS GTPase activating like protein: scaffolds CDC42 for cytoskeletal reorganization | (R. Li et al., 1999) |
| LEF1 | Lymphoid enhancing binding factor 1: transcription factor downstream of Wnt/Beta-catenin signaling | (Clevers et al., 2014) |

| MDM2 | E3 ubiquitin-protein ligase: induces proteasomal degradation of p53 , RB, IGF1R | (Girnita, Girnita, & Larsson, 2003) |
| --- | --- | --- |
| NIK | NF-kB inducing kinase: mediates activation of NF-kB in inflammation | (Neumann, Foryst-Ludwig, Klar, Schweitzer, & Naumann, 2006) |
| PIK3R1 | Phosphatidylinositol 3-kinase regulatory subunit alpha: mediates glucose uptake in adipose and skeletal muscle | (Y. T. A. Chiang et al., 2014) |
| p53 | Cellular tumor antigen p53: induce cell cycle arrest | (Kung & Murphy, 2016) |
| RAC1 | Ras-related C3 botulinum toxin substrate 1: GTPase which activates PAK1 | (Sylow et al., 2014) |
| RB1 | Retinoblastoma-associated protein: tumor suppressor which impedes G1/S phase transition | (Moreno-Navarrete et al., 2013) |
| SHC1 | SHC-transforming protein 1: adaptor which coordinates growth factor signaling pathways | (Wagner et al., 2004) |
| SOS1 | Son of sevenless homolog 1: activation of RAS | (Barroso et al., 2003) |

Supplementary Table 1*.* The pathophysiology of PAK interacting partners in T2DM.
