# Supplementary material for: p-21 Activated Kinase as a Molecular Target for Chemoprevention in Diabetes
Source: Geriatrics (Basel). 2018 Oct 19;3(4):73. doi: 10.3390/geriatrics3040073 (PMC6371191; doi:10.3390/geriatrics3040073)
Supplement: Supplementary file 1 [file geriatrics-03-00073-s001.zip › supplementary/Supplementary Figure 1_Optimize.pdf]

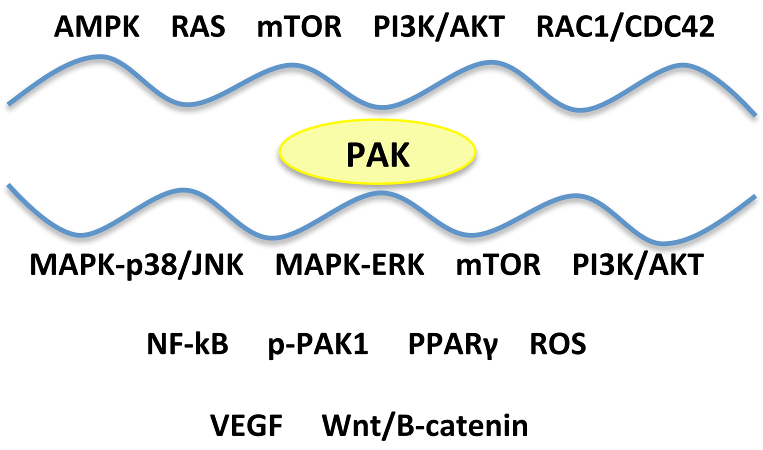

Supplementary Figure 1. Role of signaling pathways upstream and downstream of p-21 activated kinases.
