# Supplementary material for: p-21 Activated Kinase as a Molecular Target for Chemoprevention in Diabetes
Source: Geriatrics (Basel). 2018 Oct 19;3(4):73. doi: 10.3390/geriatrics3040073 (PMC6371191; doi:10.3390/geriatrics3040073)
Supplement: Supplementary file 1 [file geriatrics-03-00073-s001.zip › supplementary/Supplementary Figure legends.docx]

**Supplementary Figure 1**

**Role of signaling pathways upstream and downstream of p-21 activated kinases.** (Top) Pathways above PAK in figure are associated with PAK activation. (Bottom) PAK activation contributes to the activation of these pathways. Multiple signals lead to PAK activation via the small Rho G-proteins RAC1/CDC42, or RTK activation of membrane bound RAS. Although mTOR and PI3K/AKT pathways both converge upon PAK activation, PAK activation may also contribute to their activation. Other reports have described that AMPK signaling leads to PAK activation (Dammann et al., 2014). Depending on the cell type and environmental signal, PAK phosphorylates or scaffolds its targets and contributes to MAPK signaling, including both ERK and p38/JNK, PI3K/AKT/mTOR, NF-kB, PPAR-gamma, ROS, Wnt-Beta catenin, and VEGF (Dammann et al., 2014).

**Supplementary Figure 2**

**Diverse roles of PAK in glucose homeostasis.** Changes in PAK expression or kinase activity alter glycolysis, gluconeogenesis, and insulin release. PAK overexpression impedes glycolysis and PAK inhibition impairs gluconeogenesis and insulin release (Shalom-Barak & Knaus, 2002)(Z. Wang, Oh, & Thurmond, 2007)(Z. Wang & Thurmond, 2010).

**Supplementary Figure 3**

**PAK signaling in disease.** PAK overexpression leads to aberrant PAK signaling and drives cellular inflammation, proliferation, survival, and angiogenesis. Chronic inflammation also drives PAK signaling pathways.
