# Supplementary figures and images for: p-21 Activated Kinase as a Molecular Target for Chemoprevention in Diabetes
Source: Geriatrics (Basel). 2018 Oct 19;3(4):73. doi: 10.3390/geriatrics3040073 (PMC6371191; doi:10.3390/geriatrics3040073)

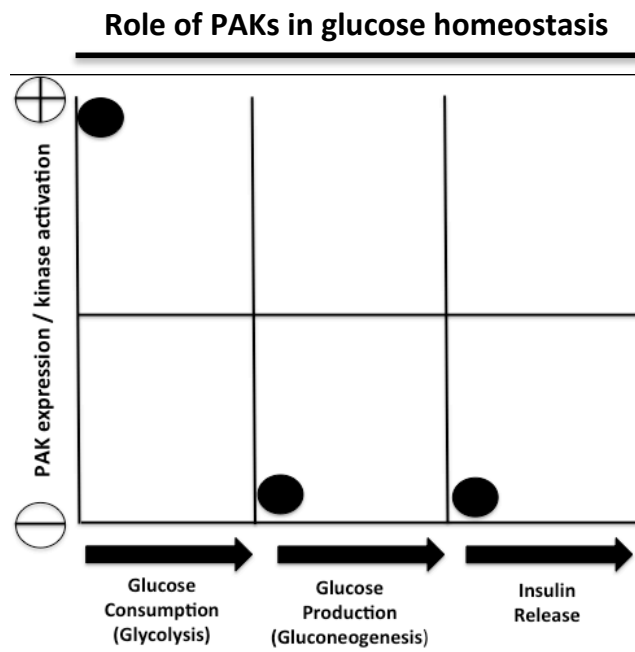

**Supplementary Figure 2. Diverse roles of PAK in glucose homeostasis.**

Supplement: Supplementary file 1 [file geriatrics-03-00073-s001.zip › supplementary/Supplementary Figure 2_Optimize.pdf]

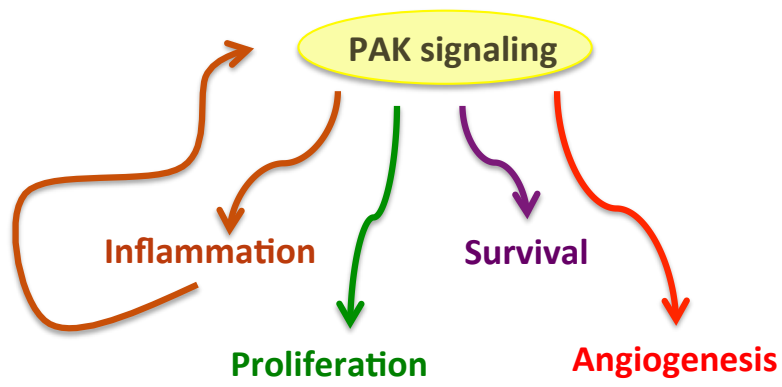

**Supplementary Figure 3. PAK signaling in disease.**

Supplement: Supplementary file 1 [file geriatrics-03-00073-s001.zip › supplementary/Supplementary Figure 3_Optimize.pdf]
